# Suggestive linkage detected for blood pressure related traits on 2q and 22q in the population on the Samoan islands

**DOI:** 10.1186/1471-2350-10-107

**Published:** 2009-10-23

**Authors:** Karolina Åberg, Feng Dai, Satupaitea Viali, John Tuitele, Guangyun Sun, Subba R Indugula, Ranjan Deka, Daniel E Weeks, Stephen T McGarvey

**Affiliations:** 1 Department of Human Genetics, Graduate School of Public Health, University of Pittsburgh, 130 Desoto St, Pittsburgh, PA 15261, USA; 2 Department of Biostatistics, Graduate School of Public Health, University of Pittsburgh, 130 Desoto St, Pittsburgh, PA 15261, USA; 3 Department of Anesthesiology, School of medicine, University of Pittsburgh, 3550 Terrace Street, Pittsburgh, PA 15261, USA; 4 Tupua Tamasese Meaole Hospital, Ministry of Health, Government of Samoa, Apia, Samoa; 5 Tafuna Family Health Center, Department of Health, American Samoa Government, Pago Pago, 96799, American Samoa; 6 Center for Genome Information, Department of Environmental Health, University of Cincinnati, Cincinnati, OH 45267, USA; 7 International Health Institute, Brown University, 121 S. Main St, Providence, RI 02912, USA; 8 Center for Biomarker Research and Personalized Medicine, School of Pharmacy, Virginia Commonwealth University, 1112 East Clay St, Richmond, VA 23298, USA

## Abstract

**Background:**

High blood pressure or hypertension is a major risk factor involved in the development of cardiovascular diseases. We conducted genome-wide variance component linkage analyses to search for loci influencing five blood pressure related traits including the quantitative traits systolic blood pressure (SBP), diastolic blood pressure (DBP) and pulse pressure (PP), the dichotomous trait hypertension (HT) and the bivariate quantitative trait SBP-DBP in families residing in American Samoa and Samoa, as well as in the combined sample from the two polities. We adjusted the traits for a number of environmental covariates such as smoking, alcohol consumption, physical activity and material life style.

**Results:**

We found suggestive univariate linkage for SBP on chromosome 2q35-q37 (LOD 2.4) and for PP on chromosome 22q13 (LOD 2.2), two chromosomal regions that recently have been associated with SBP and PP, respectively.

**Conclusion:**

We have detected additional evidence for a recently reported locus associated with SBP on chromosome 2q and a susceptibility locus for PP on chromosome 22q. However, differences observed between the results from our three partly overlapping genetically homogenous study samples from the Samoan islands suggest that additional studies should be performed in order to verify these results.

## Background

High blood pressure or hypertension is a major risk factor involved in the development of cardiovascular diseases (CVD) [1], which today are primary causes of death in most industrial countries. We have previously searched for genetic susceptibility to two other common CVD risk factors, adiposity-related phenotypes [2-4] and lipid-related phenotypes [5] in our sample from the Samoan islands. Here we continued the search for genetic susceptibility to CVD risk factors in this population by investigating blood pressure related traits. We performed genome-wide linkage investigation for a dichotomous trait for hypertension (HT) where the study subjects were divided into two categories, non-hypertensive and hypertensive as well as for three quantitative traits including systolic blood pressure (SBP), diastolic blood pressure (DBP) and pulse pressure (PP). In addition we studied the simultaneous variation of SBP and DBP (SBP-DBP) by performing a genome-wide bivariate quantitative analysis.

The population on the Samoan islands, including both American Samoa and Samoa, has a common evolutionary history of approximately 3,000 years with homogeneity of allele frequencies and linkage disequilibrium (LD) structure [6]. However, during the last century, and especially since World War II, the two polities have been differently influenced by political economic development [7-9], which has caused differences in environmental factors, such as income and wealth and nutritional behaviors, which are likely to influence the development of CVD risk factors, especially blood pressure [10-12].

Blood pressure levels and hypertension rates increase with adult age, are greater in more modernized American Samoa than in Samoa, have increased in the last 30 years, and are strongly related to adiposity measures and psychosocial stress [8,10-12]. Hypertension prevalence in 2002 and 2003 ranged from 46% in men and 31% in women from American Samoa, to 30% in men and 29% in women form Samoa [8]. Detailed studies of hypertension-related mortality have not been done in the recent decades, but earlier work suggests such mortality would be increasing since the 1970s [7].

During the last decades many genome-wide linkage investigations have been conducted for qualitative and quantitative blood pressure related traits [13] but the overlap between these studies have been small. Recently, a number of genome-wide association studies were conducted for blood pressure related traits [14-16] but so far highly significant and conclusive results have not been found. A potential reason why genome-wide linkage and association investigations of blood pressure related traits have had limited success could be attributed to the effects of varied environmental factors that are likely to influence blood pressure traits. In addition, measurement error due to the minute to minute variation in blood pressure remains a challenge in epidemiologic studies [13].

In the present study we performed genome-wide variance-component linkage analyses, as implemented in LOKI [17] and SOLAR [18,19] to search for susceptibility loci for blood pressure-related traits in American Samoa, the independent nation of Samoa, and in a combined study sample from the two polities when simultaneously taking carefully measured environmental factors into account.

## Methods

### Study Population and Samples

The study samples from the population on the Samoan islands have been extensively described elsewhere [2,3,5]. Families were selected based on maximum number of available adult family members and were collected from villages throughout American Samoa and Samoa. In total 71 families including 3016 individuals, age ≥ 18 years, were studied. The family sizes span from 2-719 individuals and the number of genotyped individuals in each family span from 2-246. As described in detail previously [3,4], the American Samoan and the Samoan samples consist of 1,465 and 1,633 genotyped and phenotyped relative pairs, respectively, useful for linkage studies. Protocols for this study were approved by the Brown University Institutional Review Board, the American Samoan Institutional Review Board, and the Government of Samoa, Ministry of Health, Health Research Committee. All participants gave their informed written consent.

### Phenotypes

Systolic (SBP) and diastolic blood pressures (DBP) were measured using mercury sphygmomanometers and the auscultatory method following American Heart Association (AHA) standard protocols. The research staff was trained using AHA audiotapes. Before the measurements were made, participants refrained from cigarette smoking for 30 minutes and emptied the bladder. Participants were seated in a quiet undisturbed place. Blood pressure was measured three times for each participating individual and the average of all measurements was used in the statistical analysis.

To include the individuals who currently were treated for hypertension, we followed the example of Hottenga et al [20], that previously was discussed by Cui et al [21] and Palmer [22], and added a factor of + 14 mmHg and + 10 mm Hg to the SBP and the DBP, respectively, for individuals receiving treatment. Pulse pressure (PP) was calculated for each individual as the differences between the average SBP and the average DBP after adjustment for treatment. For the dichotomous trait, HT, individuals who were treated for hypertension or had SBP or DBP that exceeded 140 mm Hg or 90 mm Hg, respectively, were called as hypertensive. The remaining individuals were called as non-hypertensive. Standard anthropometric techniques were used to measure stature and weight, and to calculate body mass index (BMI).

Questionnaires were used to collect self-reported information on environmental factors including education (years), moderate to heavy physical activity (hours/week), alcohol consumption (yes/no), smoking (yes/no) and material life standard. As described in our previous study [5] the information regarding material life standard was used to create a material life style index (MLSI) ranging from 1 (low material life style standard) to 12 (high material life style standard).

### Genotypes

Genotyping was performed as previously described [3,4] using the ABI PRISM linkage mapping set v2.5 MD10 (Applied Biosystems Inc., Foster City, CA). The samples from American Samoan were genotyped with an ABI PRISM 3100 genetic Analyzer and with an ABI PRISM 3130XL (Applied Biosystems Inc., Foster City, CA) while the Samoan samples were genotyped using an ABI PRISM 3130XL (Applied Biosystems Inc., Foster City, CA). In total 368 autosomal and 14 sex-linked markers were successfully genotyped in all three data sets. To be able to perform linkage analysis in the combined data set that consists of families with members from American Samoa and from Samoa that were genotyped using different genotyping platforms, we merged the genotype data according to the minimal differences in marker allele frequencies [2].

### Error Checking and Data handling

Since falsely assuming normality may lead to excessive type I errors and to a biased estimate of the major gene effect when using a variance component analysis [23] we applied Box-Cox power transformations [24] to the quantitative traits and to the covariate BMI that were not normally distributed.

As described previously [2-4] we extensively checked for genotype errors and errors of pedigree structure prior to statistical analysis using the software PEDSTATS [25], RELPAIR v2.0.1 [26,27] and PREST [28,29].

We have used the "set correct_errors 1" option in LOKI [17] to remove Mendelian inconsistent genotypes for the autosomes. For the X chromosome we used Mega2 [30] and Pedcheck [31] to remove all genotypes within the entire pedigree for a locus where a Mendelian inconsistency were detected. Mega2 and the statistical software R [32] were used to set up files needed for the analysis in this study.

### Covariate screening

To carefully take advantage of the environmental information collected from the study samples we used the "polygenic screening" command in SOLAR to investigate the significance of the covariate for each of the studied traits. Covariates with p-values ≤ 0.1 were considered as significant and only the covariates that were significant in all three study samples were included in the polygenic model. All traits from all study samples were screened for inclusion of age, sex, age*sex, age^2^, age^2 ^*sex, body mass index (BMI), cigarette smoking, alcohol consumption, education, physical activity and material life style index (MLSI). To further control for any unmeasured differences in the environment between American Samoa and Samoa that might influence the traits in the combined data set, we also screened for significance of polity of residence.

### Multipoint Linkage Analysis

In this investigation of three study samples from the Samoan islands we have applied the same statistical strategy for univariate and bivariate genome-wide linkage analysis as we previously used when investigating serum lipid levels in these study samples [5]. As done previously, we estimated marker allele frequencies from our pedigree data, while simultaneously estimating the identity-by-descent (IBD) sharing matrices using LOKI [17] and used a genetic map based on Haldane centiMorgan (cM) taken from the Rutgers Combined Linkage-Physical Map of The Human Genome [33].

#### Univariate multipoint linkage analysis

We used the multipoint variance component linkage analysis as implemented in SOLAR [18,19] to search for linkage to SBP, DBP, PP and HT on the autosomes. Prior to the actual linkage analysis we used the "polygenic -all" option in SOLAR to fit the variance components model including the covariates that were significant (p ≤ 0.10) in all three study samples. For the fitted model of a given trait vs. the null model, a likelihood-ratio test for linkage was carried out and the classical LOD scores were obtained by converting the statistic into values of log to the base 10. A LOD score ≥ 3.3 was taken as evidence of significant linkage, which is equivalent to a p-value of 0.0001 or less. A LOD score ≥ 1.9 and LOD score ≥ 1.175 were considered to show evidence of suggestive linkage and potential linkage, respectively [34]. Following the linkage analysis we used the "nplplot.R" script as implemented in the Mega2 package [30] to graph the linkage results.

Since the current version of SOLAR does not correctly carry out X-linked variance component analysis we used Mendel v7.0.0 [35] to analyze the X chromosome markers. The covariates that showed significance in the autosomal analysis were included in Mendel as predictors. We standardized the traits by gender and applied a model where X-linked QTL, random environmental, autosomal additive polygenic and X-linked additive polygenic variance components were included. Since Mendel cannot handle our largest pedigrees we broke our pedigrees into their component nuclear families using Mega2 before running Mendel.

#### Bivariate analysis

We performed genome-wide bivariate multipoint linkage analysis using SOLAR to simultaneously test for linkage to SBP and DBP (SBP-DBP). The bivariate analysis tests for linkage of two phenotypes to a single genetic region. Prior to the bivariate linkage analysis, we regressed the covariates that were significant in the univariate analysis onto the traits using a linear model. The difference between the observed and the fitted values (*i.e*. the residual) were used as the traits in the bivariate linkage investigation. The residuals appeared to be normally distributed and no further transformation was performed. We used the "loddf" command in SOLAR to transform the bivariate LOD score to 1 degree of freedom (LODeq, 1 df), which is comparable to univariate LOD score.

## Results

In the combined study sample 34% of the males and 29% of the females are hypertensive or are treated for hypertension (Table 1). Among individuals ≥ 40 years 47% of males and 45% of females are hypertensive. Hypertension levels are higher in American Samoan males than their counterparts in Samoa. We observed positive correlations of age with DBP in the younger age group, ranging from 0.24 to 0.35, while in the older age group correlations were negative among men and zero among women (Table 1). In younger adults from American Samoa and the combined sample, age and SBP correlations were generally positive. Among older women correlations between age and SBP were also positive and significant in all three study samples (Table 1).

**Table 1:** Description of Sample - non-transformed phenotypes.

	**American Samoa**		**Samoa**		**Combined**	
	**Males**	**Females**	**Males**	**Females**	**Males**	**Females**
Pedigrees^a^	34		46		71 (20^b^)	
						
Genotyped ind.^a ^(Ind. <40 years)	261 (124)	334 (160)	336 (166)	338 (137)	597 (290)	672 (297)
Age (years)^c^	43.2 (16.5)	43.0 (16.1)	41.7 (16.3)	45.2 (17.4)	42.4 (16.4)	44.1 (16.8)
Sex (%)	44	56	50	50	47	53
Education (years)^c^	11.7 (2.4)	12.0 (2.4)	9.7 (3.4)	10.0 (3.0)	10.6 (3.1)	11.0 (2.9)
Material life style index^c^	9.1 (1.8)	9.0 (1.8)	7.6 (2.6)	7.7 (2.6)	8.3 (2.4)	8.4 (2.3)
Current cigarette smokers (%)	38	20	38	13	38	17
Current alcohol use (%)	43	8	32	3	37	6
Physical activity (hours/week)^c^	4.1 (6.3)	1.9 (3.6)	8.6 (14.8)	2.3 (5.7)	6.7 (12.0)	2.1 (4.8)
Body mass index (kg/m^2^)^c^	33.5 (7.6)	36.6 (8.4)	28.9 (5.4)	33.0 (7.6)	30.9 (6.9)	34.8 (8.2)
DBP (mm Hg)^c^	85.5 (11.7)	80.6 (11.1)	82.4 (12.1)	80.1 (11.2)	83.7 (12.0)	80.3 (11.1)
SBP (mm Hg)^c^	128.9 (14.4)	123.1 (16.8)	126.4 (17.3)	122.5 (17.4)	127.4 (16.2)	122.7 (17.1)
PP (mm Hg)^c^	45.4 (11.2)	43.6 (12.2)	44.3 (11.3)	43.6 (12.6)	44.8 (11.3)	43.6 (12.4)
Correlation r - DBP vs. Age^d^	0.35^$^/-0.15*	0.25^#^/0.01	0.31^$^/-0.17*	0.24^#^/-0.04	0.32^$^/-0.16^#^	0.24^$^/-0.02
Correlation r - SBP vs. Age^d^	0.16*/-0.04	0.28^$^/0.20^#^	0.07/0.01	0.03/0.23^#^	0.11^/0.03	0.17^#^/0.21^$^
Correlation r- PP vs. Age^d^	-0.28^#^/0.21*	0.10/0.27^$^	-0.22^#^/0.21^#^	-0.20*/0.39^$^	-0.24^$^/0.21^$^	-0.04/0.32^$^
Hypertension^e ^(%)	44	30	27	28	34	29
Hypertension (%; <40 years)	34	15	17	12	24	13
Hypertension (%; ≥40 years)	57	48	38	42	47	45
Hypertensive ind. Treated (%)	32	32	13	31	20	32

^a ^Total number included. ^b ^Families including individuals from both polities. ^c ^Mean and standard error. ^d ^Pearson's correlation between phenotype and age in individuals < 40 years and ≥ 40 years, respectively. ^- p < 0.10; * - p < 0.05; ^# ^- p < 0.01; ^$ ^- p < 0.001. Calculations are made prior to correction for medication.^e^Hypertension - on anti-hypertensive medications, or SBP or DBP exceeding 140 or 90 mm Hg,

In the combined sample only 20% of males and 32% of females of all ages with hypertension are treated with antihypertensive medication (Table 1). Cigarette smoking, alcohol consumption and higher levels of physical activity are more frequently seen in males than in females. Furthermore, both males and females have remarkably high BMI (Table 1). As expected, the MLSI tends to be higher in American Samoa than in Samoa.

Heritability estimates significantly different from zero (p < 0.05) for the studied traits range from 0.11 to 0.29, where the higher values were observed for SBP and HT (Table 2). Non-significant heritability estimates (h^2^) were observed for DBP (h^2 ^= 0.08) in Samoa and for PP (h^2 ^= 0.00) in American Samoa (Table 2). The covariates included in the final model for each trait are shown in Table 2. None of the environmental covariates: education, physical activity, MLSI or polity of residence, were of significant effect for any of the investigated traits.

**Table 2:** Heritability estimates, variance and summary of significant covariates for each trait.

**Trait**	**American Samoa**				**Samoa**				**Combined sample**				**Covariates^d^**
	**N^a^**	**h^2 ^± s.e.^b^**		**Variance^c^**	**N^a^**	**h^2 ^± s.e.^b^**		**Variance^c^**	**N^a^**	**h^2 ^± s.e.^b^**		**Variance^c^**	
SBP	593	0.26	± 0.08	30%	669	0.25	± 0.08	19%	1262	0.25	± 0.06	24%	A, S, A*S, B
DBP	523	0.15	± 0.09	25%	544	0.08	*	25%	1067	0.12	± 0.06	25%	A, S, A^2^, A^2 ^*S, B, C, D
PP	595	0.00	*	12%	663	0.19	± 0.08	11%	1258	0.11	± 0.05	11%	A*S, A^2^
HT	559	0.27	± 0.15	15%	559	0.29	± 0.16	16%	1118	0.27	± 0.10	15%	A, S, B, C

^a ^Number of individuals. ^b ^Heritability estimates (h^2^) and standard error (s.e.). ^c ^Variance explained by included covariates. For the dichotomous trait, HT, the Kullback-Leibler R^2 ^is given. ^d ^Covariates included in polygenic model: A = age, A^2 ^= age^2^, S = sex, A*S = age*sex, A^2 ^*S = age^2 ^*sex, C = cigarette smoker, D = drinking alcohol and B = body mass index. * Heritability estimate is not significantly different from zero.

**Figure 1:**
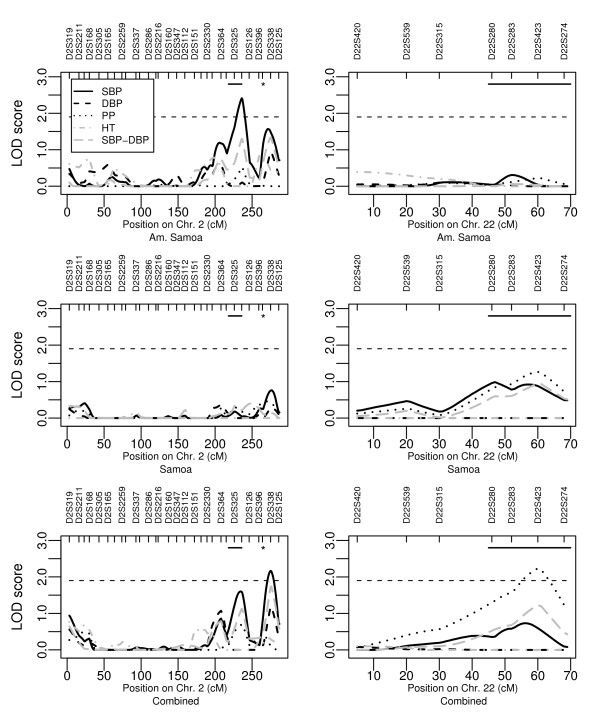
**Linkage findings for blood pressure related traits on chromosome 2 and 22**. LOD score detected on chromosome 2 (left column) and on chromosome 22 (right column) in the three study samples from the Samoan islands. The LOD score for the bivariate trait SBP-DBP was converted to 1 degree of freedom, which makes it comparable to a univariate LOD score. For chromosome 2 the approximate region where linkage with SBP previously was detected is indicated with a black horizontal line and the region where association previously was detected is indicated with an asterisk (*). For chromosome 22 the approximate region for a susceptibility locus for PP previously reported by a meta-analysis is indicated with a black horizontal line.

The strongest quantitative trait loci (QTL) detected in this study was observed for SBP (LOD = 2.41) on chromosome 2q35 in the American Samoan study sample (Figure 1 and Table 3). Approximately 40 cM from this region towards the q-telomere on chromosome 2q37 we detected another QTL with suggestive linkage (LOD ≥ 1.9) for SBP (LOD = 2.17) in the combined study sample. In this region we also detected a potential QTL for the bivariate trait SBP-DBP (LODeq = 1.7, 1 df). For PP we found a suggestive linkage signal (LOD = 2.23) on chromosome 22q13 in the combined data set (Figure 1 and Table 3). Furthermore, we detected potential linkage on chromosome 6p25 for DBP and for SBP-DBP; on chromosme 17q25 for SBP; and on chromosome 18q22 for SBP and SBP-DBP. We did not detect any signal greater than 1.5 for HT in any of the study samples. Chromosomal regions with linkage signals greater than 1.5 in at least one of the three study samples are shown in Table 3. Linkage results from all traits and chromosomes are shown in Additional file 1: Supplemental Figure S1.

**Table 3:** Chromosomal regions with maximum LOD score ≥ 1.5 (1 df) detected in at least one study sample.

**Cytogenetic Position**	**Closest marker**	**Trait**	**LOD score Am. Samoa**	**LOD score Samoa**	**LOD score Combined**
2q35	D2S2382	SBP	**2.4**	<0.5	1.6
2q37	D2S338	SBP	1.6	0.8	**2.2**
2q37	D2S338	SBP-DBP	1.3	<0.5	1.7
6p25	D6S1574	DBP	1.1	0.6	1.7
6p25	D6S1574	SBP-DBP	1.1	0.9	1.7
17q25	D17S784	SBP	<0.5	1.3	1.8
18q22	D18S61	SBP	1.1	0.7	1.8
18q22	D18S61	SBP-DBP	0.6	0.5	1.5
22q13	D22S423	PP	<0.5	1.3	**2.2**

Bold indicate suggestive linkage (LOD score ≥ 1.9).

No potential linkage (LOD ≥ 1.175) was detected for any of the traits on the X chromosome in any of the three study samples.

## Discussion

In this study, we have searched for susceptibility loci for variation in five blood pressure related traits including the quantitative traits SBP, DBP, PP, the dichotomous trait HT, and the bivariate quantitative trait SBP-DBP in three study samples from the Samoan islands.

### Accuracy of phenotypes

The phenotypes used for this study were collected by trained personnel in the participant's home environment. By collecting data in an environment familiar for the participant, we attempted to minimize the risk of a "white coat effect", where the initial blood pressure measurements are higher than the later measurements due to the stress for the individual of being in an unfamiliar environment. Our data do not indicate any overall trends in the three replicates of the blood pressure measurements and thus we have no reason to believe that a "white coat effect" influenced our data sets. Furthermore there is no "zero bias" of the measured blood pressures, meaning the last digit of the measures do not end in zero more often than expected, indicating good quality measurements without any excessive rounding.

To maximize the power of our study samples we have included all phenotyped individuals regardless of whether they were treated with antihypertensive medication or not. We adjusted for the use of antihypertensive medication by adding a factor of 14 mm Hg to the SBP and 10 mm Hg to the DBP. This is an efficient way to ensure that all available phenotypes are included in the study. However, this strategy assumes that all individuals who reported to be under antihypertensive treatment are actively using their medication and furthermore it assumes that all individuals have an equal response to their medication.

### Heritability

The heritability estimates for SBP and DBP detected for our study sample are similar to those previously reported in an earlier Samoan study population [36]. In Tokelauans, another Polynesian population, SBP and DBP heritability estimates ranged from 0.10 to 0.28 [37]. Considering the many influences on blood pressure variation, measurement issues and choice in family designs minor differences in estimates of heritability are less important than the indication that genetic variation is substantial enough to attempt identification of genomic regions.

### Susceptibility loci

In this study we identified three susceptibility loci for blood pressure related traits that reached the level of suggestive linkage. On chromosome 2q35-q37 we detected two closely placed linkage peaks for SBP with LOD score 2.4 in American Samoa and 2.2 in the combined study sample (Table 3). Within this region on chromosome 2q genome-wide significant linkage was previously found with DBP and suggestive linkage was reported with SBP [38] (Figure 1). Following these linkage findings, a recent study reported association to SBP in two independent populations on chromosome 2q37 [39] (Figure 1). Furthermore, on chromosome 22q12-q13 we detected a peak for PP with a maximum LOD score of 2.2 in the combined study sample (Table 3). This region was recently reported as the most significant locus for PP in a meta-analysis of genome-wide scans with study samples of European decent [40]. Despite the detection of two susceptibility loci on chromosome 2q and 22q in multiple studies, to our knowledge, no verified candidate genes for blood pressure related traits have yet been identified in these regions.

Recently a number of genome-wide association studies [41-48] on blood pressure related phenotypes were reported. While these studies found significant associations on multiple chromosomal regions none of them reported any major findings on chromosome 22. However, one study [43] reported significant associations with single nucleotide polymorphisms (SNPs) on chromosome 2q. Although these sequence variants are located on the same chromosomal arm as some of our findings, the closest significant marker is located approximately 25 Mbp centromeric to our linkage signal, and thus there is no direct overlap between our major linkage results and current GWAS.

### Overlap across the three study samples

The fact that the linkage signals do not overlap between the two polity-specific studies and further change when we investigate the more powerful combined study sample could be due to many factors. First, despite the homogenous population on the Samoan islands, founded by a limited numbers of individuals and fairly isolated for approximately 3,000 years, the power to detect linkage for complex traits within this study sample is limited. Thus the lack of overlap between the study samples suggests that studies of such complex traits as blood pressure related traits require a further extended study sample to detect significant linkage. Furthermore, although the study samples are from a genetically homogenous population, the environmental exposure is different between the American Samoan and the Samoan study sample as well as within the two polity-specific samples. Substantial variation in environmental influences might cause fluctuations in the investigated traits that are far larger than the effect of the genetic component/s and therefore no highly significant genetic susceptibility can be detected. In an attempt to control for alterations of environmental factors we have screened for inclusion of different environmental covariates including cigarette smoking, alcohol consumption, physical activity, education and material life style index. Somewhat surprisingly, none of the environmental factors showed significant effects on SBP, PP or HT and only cigarette smoking and alcohol consumption were of significant effect for DBP in all three study samples, which may suggest that physical covariates such as age, sex and BMI are of greater importance for blood pressure related traits than social and behavioral environmental factors.

The overlap in genomic regions identified between our previous studies on adiposity- and lipid-related phenotypes and our current study is negligible [2-6]. Taken together these results may suggest that no common chromosomal regions with major effects for the metabolic syndrome exist within the studied population. However, considering the different levels of heritability detected for the different phenotypes in the study samples, our statistical power to detect shared genetic effects may be limited. Future work remains to explicitly test for pleiotropy across metabolic, lipid and cardiovascular phenotypes in the Samoan study sample.

## Conclusion

In this study we have detected additional evidence for a recently reported susceptibility loci for SBP on chromosome 2q and a susceptibility locus for PP on chromosome 22q. However, the differences observed in the results from the three investigated study samples from the Samoan islands suggest additional studies should be performed in order to further verify these results.

## Authors' contributions

KÅ, mentored by DEW, carried out the statistical analyses of the data and wrote the manuscript. FD, under the supervision of DEW, performed checking and correction of reported pedigrees and wrote the original scripts for data checking and bivariate linkage analysis. SV and JT provided support and advice for the fieldwork in Samoa and American Samoa, respectively. GS and SRI conducted genotyping of all samples. STM, RD, and DEW collaboratively designed and led this study and obtained funding for it. All the authors read and approved the final manuscript.

## Pre-publication history

The pre-publication history for this paper can be accessed here:



## Supplementary Material

Additional file 1 **Plots of genome-wide LOD scores of blood pressure related traits.** Click here for file
