# Supplementary material for: Suggestive linkage detected for blood pressure related traits on 2q and 22q in the population on the Samoan islands
Source: BMC Med Genet. 2009 Oct 23;10:107. doi: 10.1186/1471-2350-10-107 (PMC2770055; doi:10.1186/1471-2350-10-107)
Supplement: Additional file 1 — Plots of genome-wide LOD scores of blood pressure related traits. [file 1471-2350-10-107-S1.PDF]

Supplementary material to:

Suggestive linkage detected for blood pressure related traits on 2q and 22q in the population on the Samoan islands

Karolina Åberg, Feng Dai, Satupaitea Viali, John Tuitele, Guangyun Sun, Subba Rao Indugula, Ranjan Deka, Daniel E. Weeks, Stephen T. McGarvey *

*Correspondence to:
Dr. Stephen T. McGarvey, PhD, MPH
Professor of Community Health and Anthropology
Director, International Health Institute
Brown University
Box G- S2
169 Angell Street, Room 200
Providence, RI 02912
phone: 401-863-1354
fax: 401-863-1243
email: Stephen_McGarvey@brown.edu

Supplementary Figure S1. Genome-wide LOD score detected for five blood pressure related traits in American Samoa (left column), Samoa (middle column) and in the combined study sample (right column). The LOD score for the bivariate trait SBP-DBP was converted to 1 degree of freedom, which makes it comparable to a univariate LOD score.

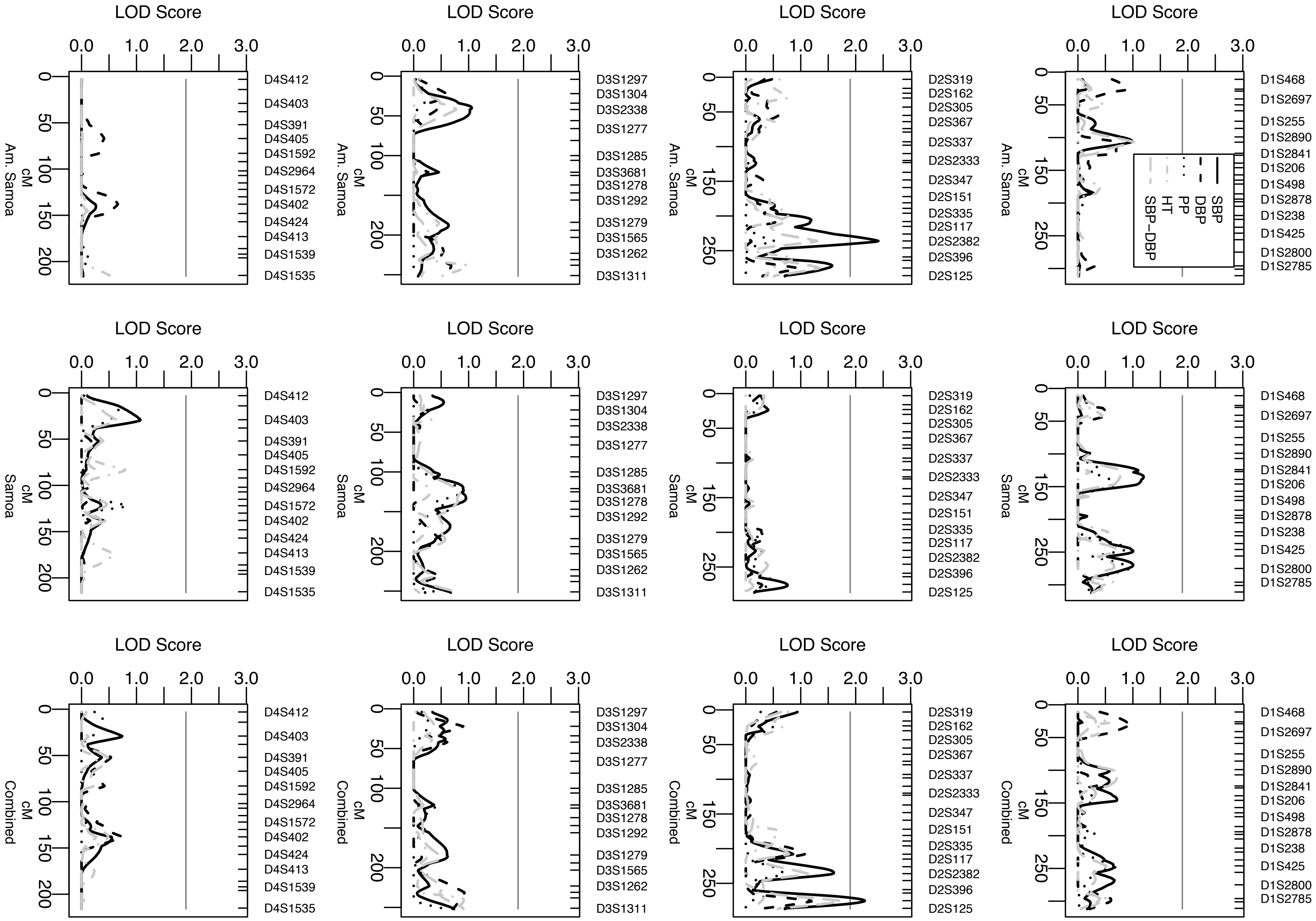

LOD Score
0.0 1.0 2.0 3.0
cM
Am. Samoa
Samoa
Combined
D4S412
D4S403
D4S391
D4S405
D4S1592
D4S2964
D4S1572
D4S402
D4S424
D4S413
D4S1539
D4S1535
D3S1297
D3S1304
D3S2338
D3S1277
D3S1285
D3S3681
D3S1278
D3S1292
D3S1279
D3S1565
D3S1262
D3S1311
D2S319
D2S162
D2S305
D2S367
D2S337
D2S2333
D2S347
D2S151
D2S335
D2S117
D2S2382
D2S396
D2S125
D1S468
D1S2697
D1S255
D1S2890
D1S2841
D1S206
D1S498
D1S2878
D1S238
D1S425
D1S2800
D1S2785
SBP
DBP
PP
HT
SBP−DBP

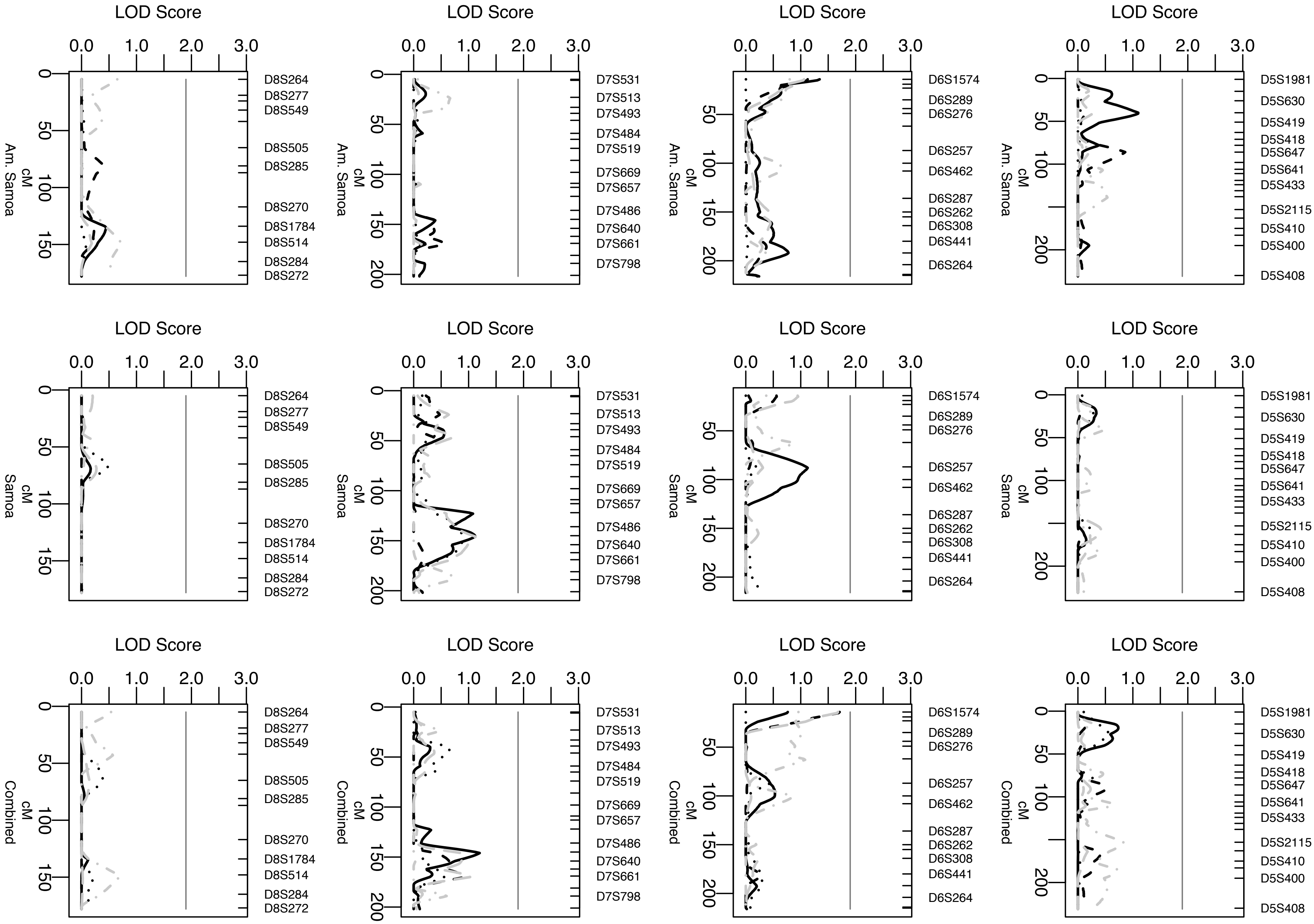
LOD Score
0.0 1.0 2.0 3.0
cM
Am. Samoa
Samoa
Combined
D8S264
D8S277
D8S549
D8S505
D8S285
D8S270
D8S1784
D8S514
D8S284
D8S272
D7S531
D7S513
D7S493
D7S484
D7S519
D7S669
D7S657
D7S486
D7S640
D7S661
D7S798
D6S1574
D6S289
D6S276
D6S257
D6S462
D6S287
D6S262
D6S308
D6S441
D6S264
D5S1981
D5S630
D5S419
D5S418
D5S647
D5S641
D5S433
D5S2115
D5S410
D5S400
D5S408

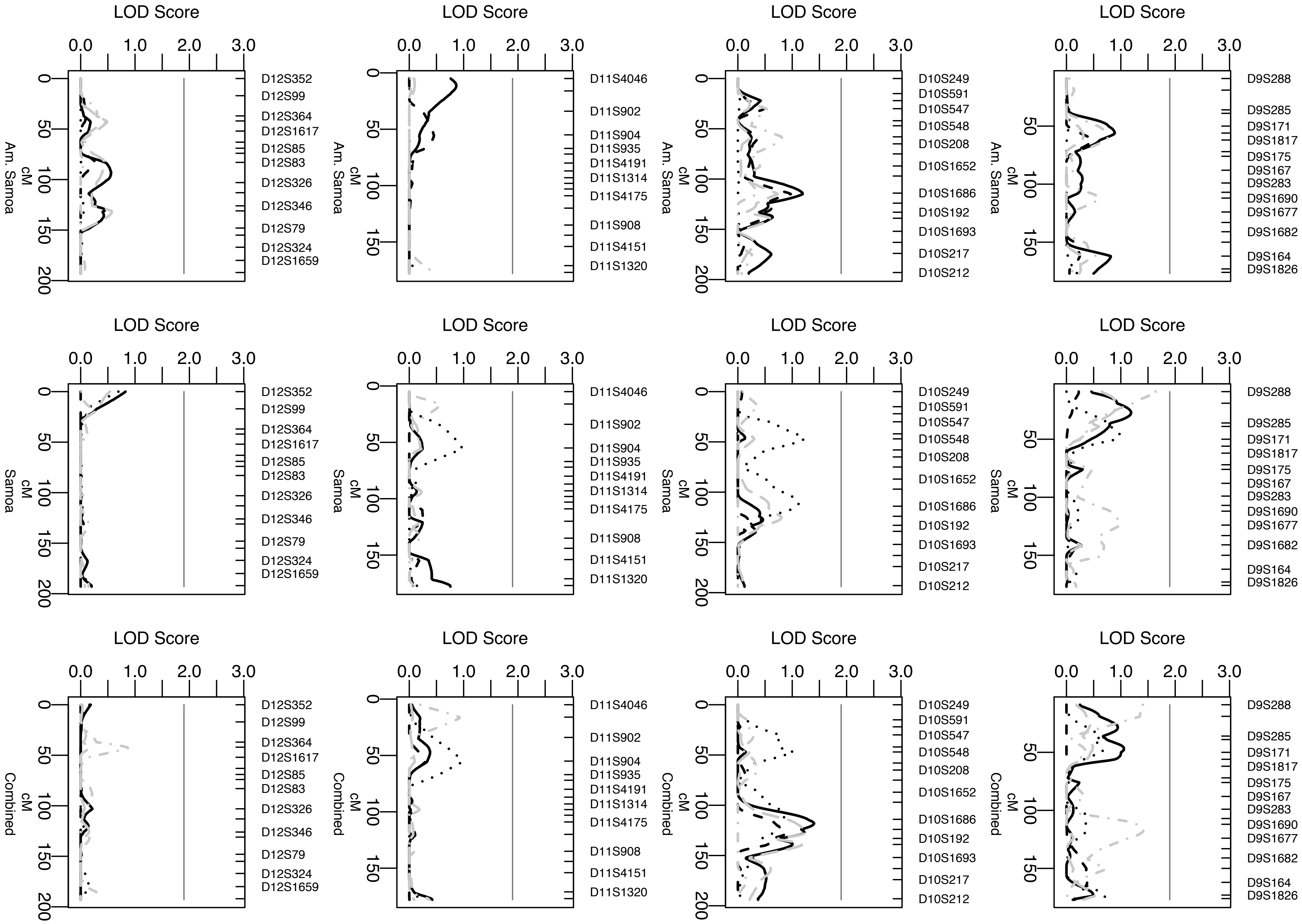
LOD Score
0.0
1.0
2.0
3.0
cM
Am. Samoa
Samoa
Combined
0
50
100
150
200
D12S352
D12S99
D12S364
D12S1617
D12S85
D12S83
D12S326
D12S346
D12S79
D12S324
D12S1659
D11S4046
D11S902
D11S904
D11S935
D11S4191
D11S1314
D11S4175
D11S908
D11S4151
D11S1320
D10S249
D10S591
D10S547
D10S548
D10S208
D10S1652
D10S1686
D10S192
D10S1693
D10S217
D10S212
D9S288
D9S285
D9S171
D9S1817
D9S175
D9S167
D9S283
D9S1690
D9S1677
D9S1682
D9S164
D9S1826

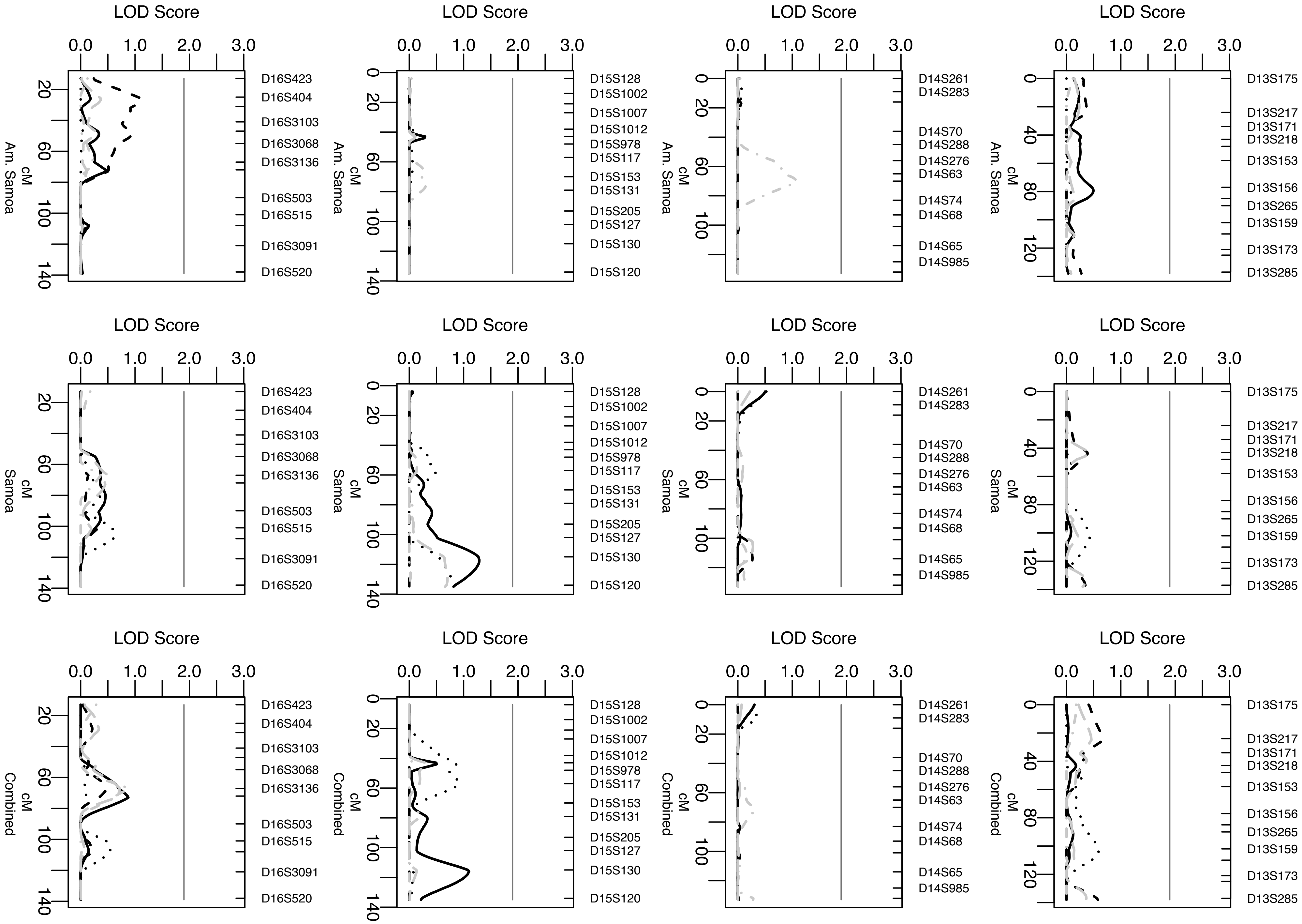
LOD Score
0.0 1.0 2.0 3.0
cM
Am. Samoa
Samoa
Combined
D16S423
D16S404
D16S3103
D16S3068
D16S3136
D16S503
D16S515
D16S3091
D16S520
D15S128
D15S1002
D15S1007
D15S1012
D15S978
D15S117
D15S153
D15S131
D15S205
D15S127
D15S130
D15S120
D14S261
D14S283
D14S70
D14S288
D14S276
D14S63
D14S74
D14S68
D14S65
D14S985
D13S175
D13S217
D13S171
D13S218
D13S153
D13S156
D13S265
D13S159
D13S173
D13S285
0 20 40 60 80 100 120 140

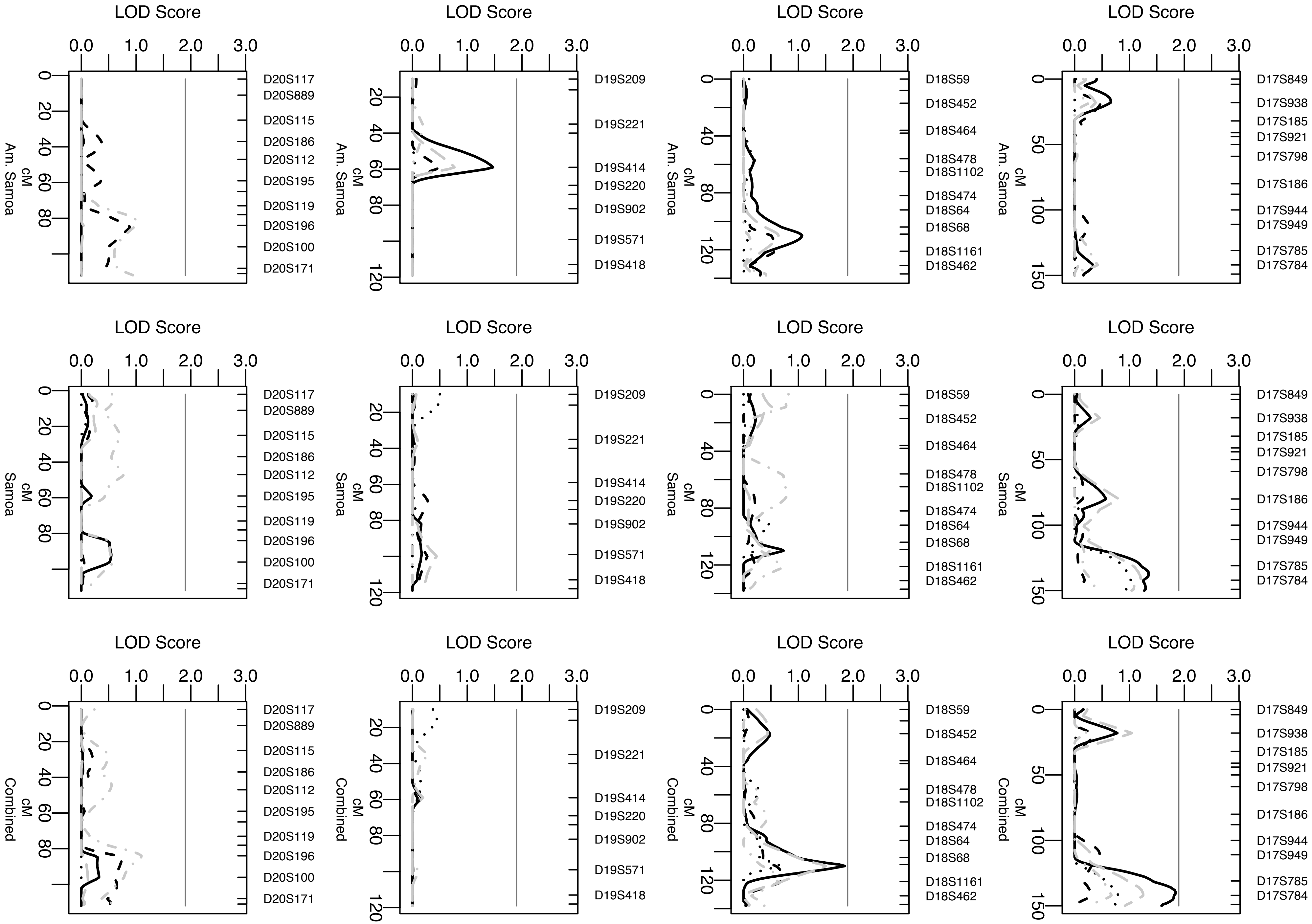
LOD Score
0.0
1.0
2.0
3.0
cM
Am. Samoa
Samoa
Combined
D20S117
D20S889
D20S115
D20S186
D20S112
D20S195
D20S119
D20S196
D20S100
D20S171
D19S209
D19S221
D19S414
D19S220
D19S902
D19S571
D19S418
D18S59
D18S452
D18S464
D18S478
D18S1102
D18S474
D18S64
D18S68
D18S1161
D18S462
D17S849
D17S938
D17S185
D17S921
D17S798
D17S186
D17S944
D17S949
D17S785
D17S784

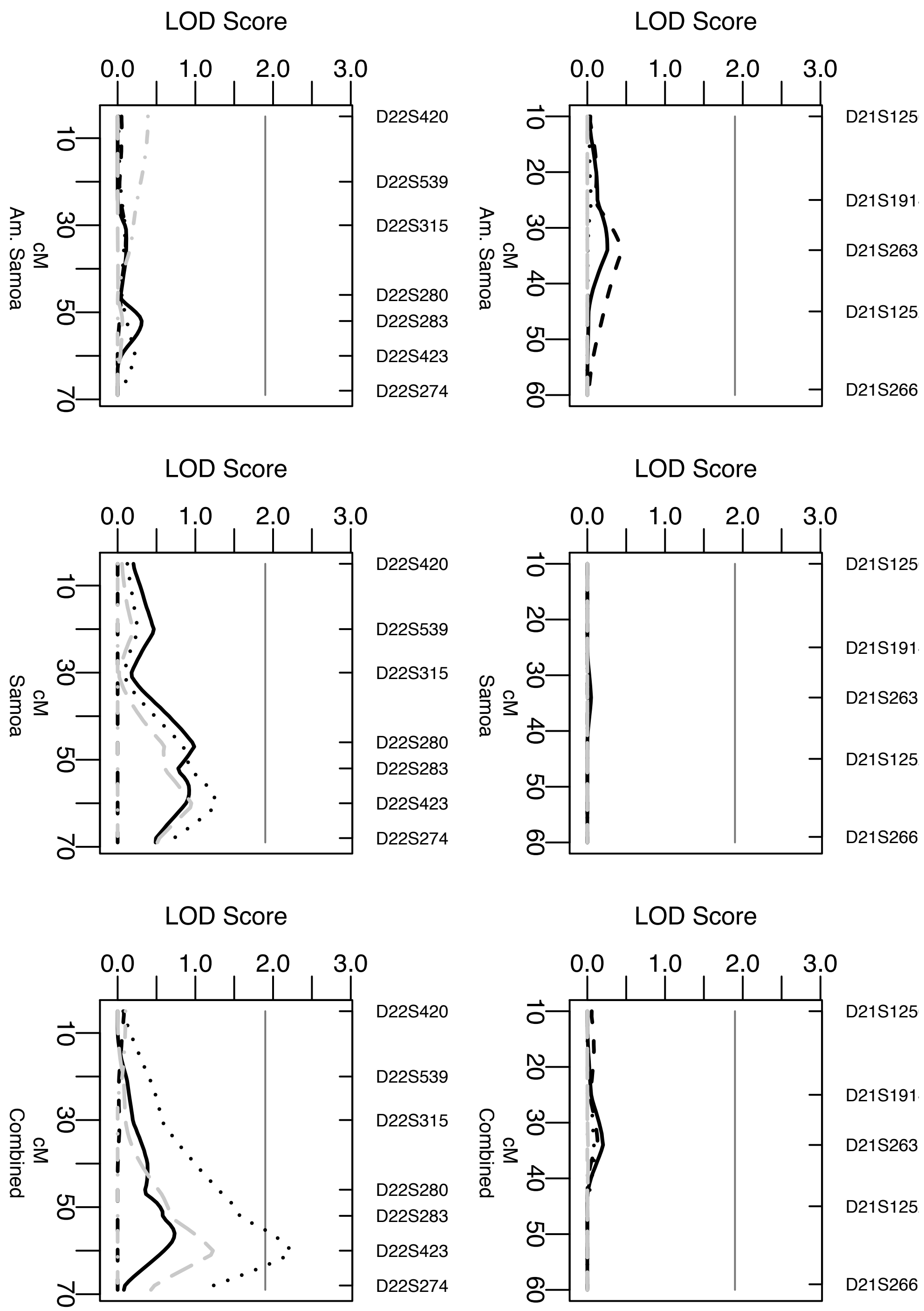

LOD Score
0.0
1.0
2.0
3.0
cM
Am. Samoa
Samoa
Combined
D22S420
D22S539
D22S315
D22S280
D22S283
D22S423
D22S274
D21S125
D21S191
D21S263
D21S266
